# Perfusion parameters of triphasic computed tomography hold preoperative prediction value for microvascular invasion in hepatocellular carcinoma

**DOI:** 10.1038/s41598-023-35913-y

**Published:** 2023-05-27

**Authors:** Li Zhang, Guodong Pang, Jing Zhang, Zhenguo Yuan

**Affiliations:** 1 Department of Radiology, The Second Hospital, Cheeloo College of Medicine, Shandong University, Jinan, 250033 Shandong China; 2 Department of Radiology, Shandong Provincial Hospital, Cheeloo College of Medicine, Shandong University, Jinan, 250021 Shandong China; 3 Department of Radiology, Shandong Provincial Hospital Affiliated to Shandong First Medical University, Jinan, 250021 Shandong China

**Keywords:** Cancer, Diseases

## Abstract

The purpose of this study was to evaluate perfusion parameters of triphasic computed tomography (CT) scans in predicting microvascular invasion (MVI) in hepatocellular carcinoma (HCC). All patients were pathologically diagnosed as HCC and underwent triple-phase enhanced CT imaging, which was used to calculate the blood perfusion parameters of hepatic arterial supply perfusion (HAP), portal vein blood supply perfusion (PVP), hepatic artery perfusion Index (HPI), and arterial enhancement fraction (AEF). Receiver operating characteristic (ROC) curve was used to evaluate the performance. The mean values of PVP(Min), AEF(Min), the difference in PVP, HPI and AEF related parameters, the relative PVP(Min) and AEF(Min) in MVI negative group were significantly higher than those in MVI positive group, while for the difference in HPI(Max), the relative HPI(Max) and AEF(Max), the value of MVI positive group significantly higher than that of negative group. The combination of PVP, HPI and AEF had the highest diagnostic efficacy. The two parameters related to HPI had the highest sensitivity, while the combination of PVP related parameters had higher specificity. A combination of perfusion parameters in patients with HCC derived from traditional triphasic CT scans can be used as a preoperative biomarker for predicting MVI.

## Introduction

Hepatocellular carcinoma (HCC) is the sixth most common cancer worldwide and has a high mortality rate^1^. Radical treatments of HCC include liver resection, liver transplantation and ablation, as the first-choice treatments for patients with early-stage HCC^2^. However, the 5-year tumor recurrence and metastasis rate after liver resection is as high as 40 to 70%, which seriously affect patients’ prognosis^3,4^. Microvascular invasion (MVI) is an independent risk factor associated with recurrence and poor overall survival of HCC as reported in previous studies^5,6^. It serves as an important reference for evaluating recurrence risks and selecting treatment options. A meta-analysis indicated that MVI was correlated with reduced 5-year disease free survival (DFS) rates^7^. Even for isolated HCC smaller than 2 cm, overall survival (OS) and recurrence free survival (RFS) in patients with MVI is still lower than in patients without MVI^8,9^.

MVI is defined by the observation of cancer cell nests in the vascular lumen lined by endothelial cells^10,11^. However, one limitation of MVI is that it can only be diagnosed pathologically under the microscope after surgery. Earlier studies have shown that MVI is related to poor HCC prognosis^12^, and is critical to the choice of preoperative HCC treatment options. Therefore, preoperative diagnosis of MVI is very important. Fortunately, in recent years, research has progressed in predicting MVI before surgery. Computed tomography (CT) and magnetic resonance imaging (MRI), especially gadoxetate-enhanced MRI, have significant potential in predicting MVI. Radiological features such as large lesion size, non-smooth tumor margins, arterial peritumoral enhancement, tumor hypointensity or peritumoral hypointensity on hepatobiliary phase (HBP), non-enhancing capsule, can be used as biomarkers to predict MVI preoperatively^13–17^. However, these radiological features are, to a certain degree, subjective and lack external validation. Radiomics, a relatively new discipline, has also been used by some scholars to predict MVI for HCC^18–20^. Radiomics refers to the extraction of large amounts of imaging information to aid in the diagnosis and treatment of diseases^14^. Nevertheless, the reproducibility of radiomics is poor, though it is more objective.

Perfusion computed tomography (PCT) can be used to quantitatively measure liver perfusion parameters and therefore be applied for liver perfusion evaluation. It was first conceptualized by Miles et al. and is considered to be a valuable tool for reflecting hepatic hemodynamics^21^. However, traditional PCT generally has a high radiation dose, which makes it difficult to be widely used in clinical practice. Blomley et al.^22^ first proposed that standard triphasic CT with a dual maximal slope model can be used to obtain perfusion parameters and reflect blood flow of the lesions similar to a full PCT dataset.

To our knowledge, limited studies have reported the investigation of PCT in predicating MVI of HCC. Thereby, the purpose of the current study is to research the predicative value of PCT parameters for MVI with the hope of obtaining relatively objective and reproducible biomarkers for prognosis in HCC prior to surgery.

## Materials and methods

The study was approved by the Ethics Committee of the Second Hospital of Shandong University (No. KYLL-2022LW169). The need to obtain informed consent from patients for our study was waived by the Ethics Committee of the Second Hospital of Shandong University. Consent for CT scanning and surgery had been obtained from all participants in the study. The research methods were conducted in accordance with the declaration of Helsinki.

### Patients

81 patients were enrolled in the study and preoperative CT was performed from February 2016 to March 2020. Detailed patients’ information can be found in Table 1.

**Table 1:** Comparisons of patient characteristics.

Characteristic	MVI-negative (n = 33)	MVI-positive (n = 48)	P value
Age (years)^a^	54.97± 10.93	57.29± 9.58	0.315
Sex (male/female)	29/4	42/6	0.620
HBV/non-HBV	29/4	43/5	0.540
AFP (>20 ng/mL)	12/21	28/20	0.052
Diameter (cm)	3.40±2.14	5.52±2.86	0.001

P value: comparison between MVI negative and positive patients.

^a^Data are means ± standard deviations.

**Figure 1:**
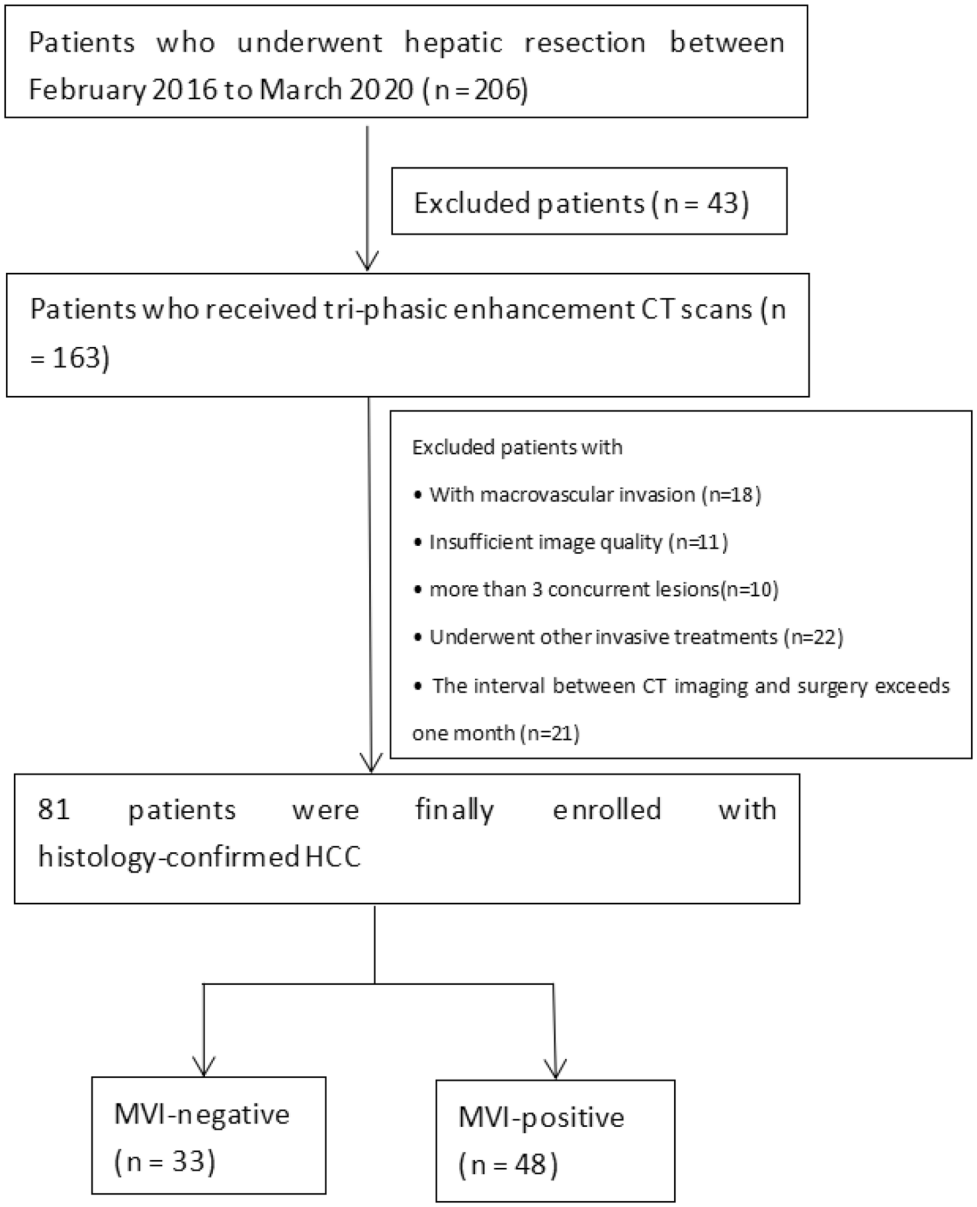
Flow chart of inclusion and exclusion criteria for the subject enrollment in the study. *CT* computed tomography, *HCC* hepatocellular carcinoma, *MVI* microvascular invasion.

A flow diagram of inclusion and exclusion criteria is shown in Fig. 1. Briefly, inclusion criteria were as follows: (1) histopathologically diagnosed as HCC; (2) No history of liver surgery and other invasive treatments; (3) triphasic enhancement CT scan was performed; (4) preoperative CT examination was performed less than 1 month before surgery.

#### Pathological diagnostic criteria

Using the “7 points” baseline sampling method for specimen sampling^23^. MVI refers to the observation of cancer cell nests in the lumen of blood vessels lined with endothelial cells under a microscope, mainly consisting of branches of the paracancerous portal vein (including blood vessels within the capsule).

### Computed tomography (CT) protocol

Breathing training before scanning. CT examinations were performed on GE Discovery CT750 HD (GE Healthcare, Chicago, IL, US). Tri-phasic enhancement CT scan was performed. Contrast agent (Visipaque 270mg iodine/ml; GE Healthcare, Chicago, IL, US) was administered for a total dose of 100 ml, followed by a 30-ml saline flush at a rate of 3.5–4.0 mL/s. The arterial phase CT scan was performed at 30–35s after the injection of contrast agent. Portal venous phase CT scan and equilibrium phase CT scan was performed 60–70s and 3 min post-injection, respectively.

The scan parameters were as follows: 120kV tube voltage, 200 mAs tube current, 1.375 pitch, 5 mm layer thickness and 2 mm reconstruction layer interval.

### Perfusion parameter measurements

**Figure 2:**
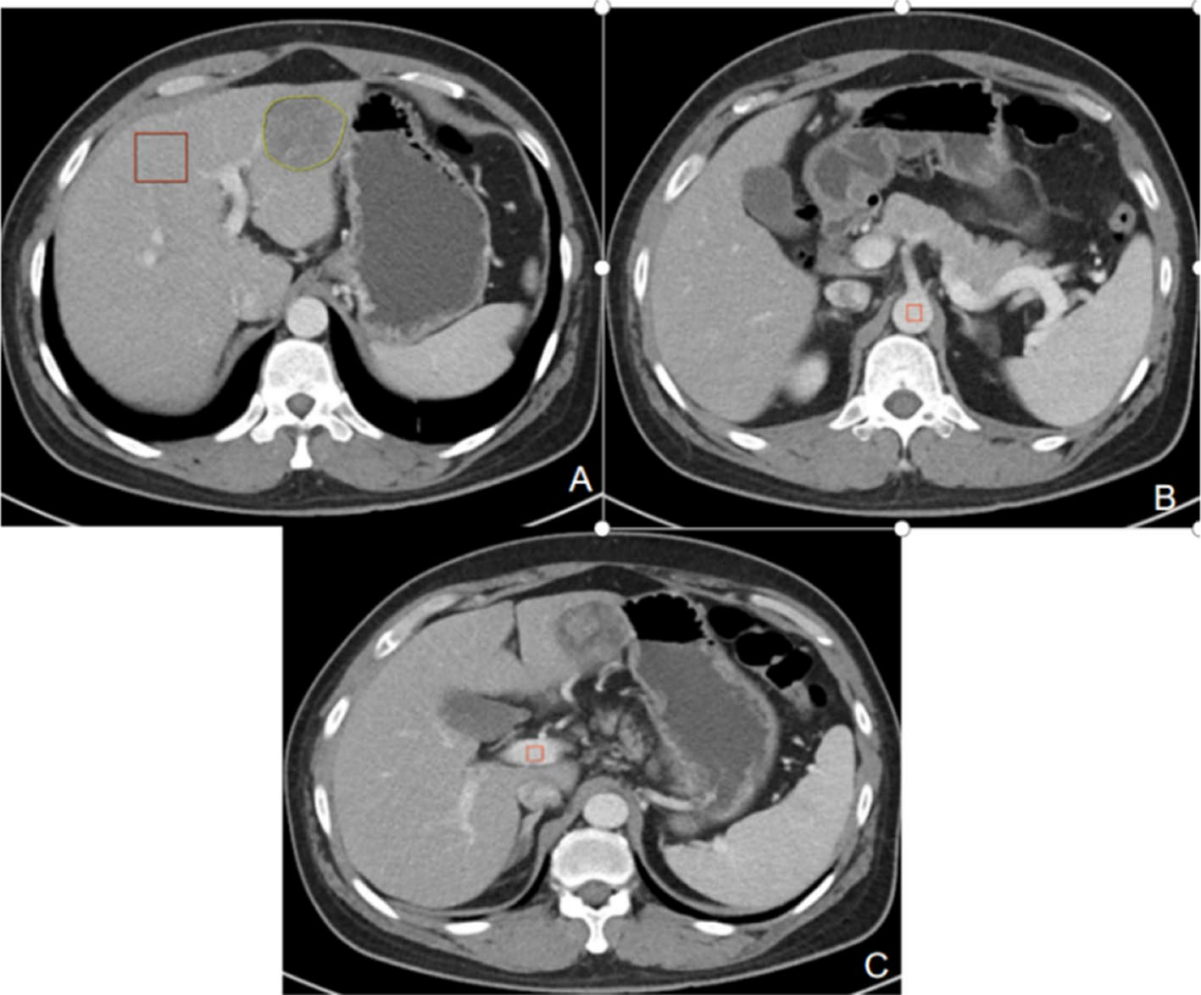
ROI setting was shown. The area delineated by the yellow line in Figure (**A**) is the lesion, while the red line represents the surrounding normal liver parenchyma. The red lines in Figures (**B**) and (**C**) depict the abdominal aorta and portal vein, respectively.

Load images into CT hemodynamic kinetics software (GE Healthcare, Chicago, IL, US). Draw the regions of interest (ROIs) on the images by two radiologists with more than 3 years of work experience. For patients with non-solitary lesions, the largest lesion was selected as the target lesion.The ROIs included the lesion and its size-matched normal liver parenchyma, the abdominal aorta (at the level of the celiac artery), and the portal vein (near the bifurcation). Larger blood vessels in the liver were avoided when drawing the ROI. ROI setting was shown in Fig. 2. Tri-phasic CT scan based on model-free maximum was used to calculate the perfusion parameters of hepatic arterial supply perfusion (HAP), portal vein blood supply perfusion (PVP), hepatic artery perfusion Index (HPI), and arterial enhancement fraction (AEF). Other perfusion parameters are then be calculated: the difference in HAP(ΔHAP = HAP_tumor_ − HAP_liver_), relative HAP (rHAP = ΔHAP/HAP_liver_), the difference in PVP (ΔPVP = PVP _tumor_− PVP_liver_), relative PVP (rPVP = ΔPVP/PVP_liver_), the difference in HPI (ΔHPI = HPI_tumor_ − HPI_liver_), relative HPI (rHPI = ΔHPI/HPI_liver_), the difference in AEF (ΔAEF = AEF_tumor_ − AEF_liver_), and the relative AEF (rAEF = ΔAEF/AEF_liver_).

### Statistical analysis

All Statistical analyses were performed using SPSS software (SPSS statistics; IBM). Bilateral P values less than 0.05 are considered significant differences. The T test was used to analyze continuous variables, while Chi-square test was used for analysis of categorical variables. The data of the perfusion parameters were non-normally distributed, so the Mann-Whitney *U* test was used. Receiver operating characteristic (ROC) curves were used to reflect the potential diagnostic performance of the liver perfusion parameter and were chosen to calculate the sensitivity, specificity and cut-off value.

## Results

### The clinical characteristics

A total of 81 HCC patients were enrolled in the study, with a total of 89 lesions. Sex ratio was 71:10, male to female, and mean age was 56.34 years old with a range of 31–81. A history of HBV infection was reported in 72 patients, while the other 9 cases had no HBV infection. Lesion diameters ranged from 1.0 to 14.0 cm with a mean diameter of 4.6 cm. Among all included patients, 33 cases were MVI negative and 48 cases were MVI positive.

Among 33 MVI negative patients, mean age was 54.97 years old, compared to 57.29 years old for the 48 MVI positive patients. Sex ratio (male to female) was 29:4 and 42:6, respectively, for MVI negative and positive patients. 29 out of 33 MVI negative and 43 out of 48 MVI positive patients reported HBV history. 12 of the MVI negative patients had alpha fetoprotein (AFP) levels higher than 20 ng/mL, while 28 of the MVI positive patients had AFP levels higher than 20 ng/mL. The mean lesion diameter for MVI negative patients was 3.40 cm, while the mean lesion diameter for MVI positive patients was 5.52 cm. Between the two groups, significant differences were only found in lesion diameters, but not in age, sex, AFP and HBV history. Summary of clinical characteristics is shown in Table 1.

### Correlation of perfusion parameters with the MVI of HCC

**Figure 3:**
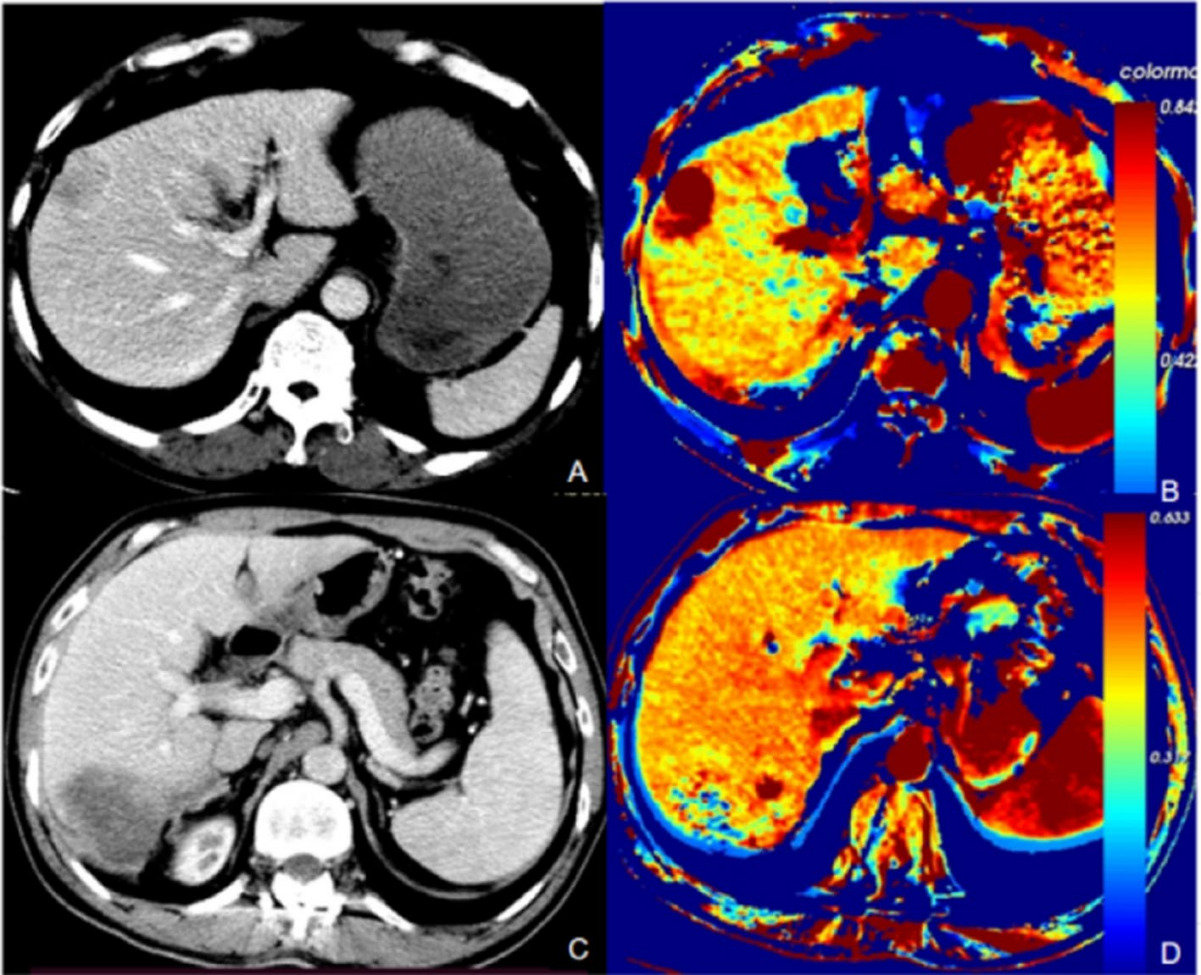
Quantitative parameter images derived from the triphasic CT scans. (**A**) and (**C**) are portal phase images of MVI negative and positive patients. (**B**) and (**D**) are corresponding perfusion images (AEF). The value of AEF in MVI negative patient was significantly higher than in MVI positive patient.

The perfusion parameters are shown in Tables 2, 3 and 4. For the perfusion parameters calculated by the software directly, the mean values of PVP(Min) and AEF(Min) in MVI negative group were 0.107 and 0.295, significantly higher than 0.042 and 0.158 in MVI positive group with P values of 0.035 and 0.005 respectively (Table 2). Quantitative parameter images derived from the triphasic CT scans of MVI negative and positive patients are shown in Fig. 3.

**Table 2:** Liver perfusion parameters in patients with MVI-negative and MVI-positive.

Group	Mean value ± SD				P value
	MVI-negative (n = 33)	MVI-positive (n = 48)	MVI-negative (n = 33)	MVI-positive (n = 48)	
PVP(Min)	0.107	0.042	0.134	0.097	0.035*
PVP(Max)	0.534	0.586	0.198	0.208	0.301
PVP(Median)	0.287	0.258	0.115	0.105	0.124
PVP(Mean)	0.289	0.263	0.115	0.093	0.122
PVP(0.1)	0.192	0.157	0.129	0.106	0.194
PVP(0.25)	0.228	0.201	0.127	0.107	0.265
PVP(0.5)	0.286	0.258	0.115	0.105	0.152
PVP(0.75)	0.336	0.317	0.124	0.102	0.144
PVP(0.9)	0.383	0.371	0.143	0.116	0.299
HPI(Min)	0.385	0.345	0.052	0.117	0.242
HPI(Max)	0.478	0.498	0.040	0.025	0.056
HPI(Median)	0.434	0.442	0.033	0.028	0.107
HPI(Mean)	0.421	0.441	0.077	0.026	0.076
HPI(0.1)	0.412	0.415	0.038	0.038	0.214
HPI(0.25)	0.423	0.428	0.035	0.031	0.146
HPI(0.5)	0.436	0.442	0.035	0.028	0.172
HPI(0.75)	0.448	0.454	0.036	0.026	0.381
HPI(0.9)	0.455	0.466	0.036	0.026	0.165
AEF(Min)	0.295	0.158	0.217	0.194	0.005*
AEF(Max)	1.902	2.230	1.746	1.913	0.231
AEF(Median)	0.593	0.513	0.175	0.159	0.052
AEF(Mean)	0.605	0.531	0.179	0.142	0.119
AEF(0.1)	0.451	0.375	0.195	0.204	0.106
AEF(0.25)	0.506	0.447	0.197	0.177	0.201
AEF(0.5)	0.592	0.513	0.175	0.158	0.066
AEF(0.75)	0.656	0.585	0.218	0.124	0.102
AEF(0.9)	0.755	0.656	0.274	0.144	0.304

^*^Statistically significant difference between the two groups (P < 0.05).

**Table 3:** The difference and relative liver perfusion parameters in patients with MVI-negative and MVI-positive.

Group	Mean value ± SD				P value
	MVI-negative (n = 33)	MVI-positive (n = 48)	MVI-negative (n = 33)	MVI-positive (n = 48)	
ΔPVP(Min)	− 0.163	− 0.276	0.17	0.151	0.012*
ΔPVP(Max)	0.107	0.142	0.193	0.17	0.223
ΔPVP(Median)	− 0.057	− 0.125	0.121	0.116	0.028*
ΔPVP(Mean)	− 0.057	− 0.119	0.115	0.109	0.023*
ΔPVP(0.1)	− 0.107	− 0.187	0.155	0.143	0.044*
ΔPVP(0.25)	− 0.094	− 0.161	0.137	0.142	0.062
ΔPVP(0.5)	− 0.058	− 0.124	0.123	0.113	0.031*
ΔPVP(0.75)	− 0.036	− 0.084	0.116	0.103	0.062
ΔPVP(0.9)	− 0.013	− 0.047	0.126	0.947	0.146
ΔHPI(Min)	− 0.016	− 0.054	0.033	0.104	0.058
ΔHPI(Max)	0.044	0.072	0.052	0.044	0.019*
ΔHPI(Median)	0.016	0.031	0.032	0.029	0.060
ΔHPI(Mean)	0.003	0.029	0.089	0.026	0.061
ΔHPI(0.1)	0.004	0.011	0.027	0.018	0.185
ΔHPI(0.25)	0.010	0.020	0.028	0.023	0.096
ΔHPI(0.5)	0.017	0.030	0.030	0.029	0.072
ΔHPI(0.75)	0.025	0.038	0.035	0.034	0.085
ΔHPI(0.9)	0.013	0.046	0.101	0.038	0.065
ΔAEF(Min)	− 0.086	− 0.232	0.223	0.194	0.005*
ΔAEF(Max)	1.284	1.744	1.951	1.894	0.075
ΔAEF(Median)	0.120	0.076	0.149	0.121	0.501
ΔAEF(Mean)	0.134	0.095	0.171	0.101	0.408
ΔAEF(0.1)	0.022	− 0.033	0.176	0.159	0.219
ΔAEF(0.25)	0.051	0.025	0.174	0.125	0.448
ΔAEF(0.5)	0.119	0.076	0.149	0.120	0.564
ΔAEF(0.75)	0.165	0.134	0.194	0.110	0.848
ΔAEF(0.9)	0.250	0.192	0.282	0.128	0.751

**Table 4:** The difference and relative liver perfusion parameters in patients with MVI-negative and MVI-positive.

Group	Mean value ± SD				P value
	MVI-negative (n = 33)	MVI-positive (n = 48)	MVI-negative (n = 33)	MVI-positive (n = 48)	
rPVP(Min)	− 0.664	− 0.862	0.369	0.333	0.035*
rPVP(Max)	0.304	0.312	0.51	0.355	0.441
rPVP(Median)	− 0.173	− 0.300	0.298	0.265	0.076
rPVP(Mean)	− 0.172	− 0.293	0.278	0.24	0.057
rPVP(0.1)	− 0.403	− 0.513	0.342	0.324	0.135
rPVP(0.25)	− 0.315	− 0.407	0.326	0.313	0.155
rPVP(0.5)	− 0.174	− 0.299	0.299	0.266	0.079
rPVP(0.75)	− 0.086	− 0.201	0.303	0.226	0.127
rPVP(0.9)	− 0.008	− 0.109	0.333	0.205	0.158
rHPI(Min)	− 0.040	− 0.139	0.083	0.271	0.061
rHPI(Max)	0.110	0.179	0.126	0.132	0.021*
rHPI(Median)	0.432	0.080	0.079	0.095	0.079
rHPI(Mean)	0.182	0.077	0.186	0.083	0.079
rHPI(0.1)	0.013	0.031	0.065	0.057	0.208
rHPI(0.25)	0.028	0.056	0.068	0.076	0.111
rHPI(0.5)	0.046	0.081	0.075	0.096	0.096
rHPI(0.75)	0.064	0.099	0.088	0.103	0.098
rHPI(0.9)	0.042	0.119	0.211	0.112	0.058
rAEF(Min)	− 0.287	− 0.612	0.473	0.456	0.008*
rAEF(Max)	2.450	3.675	3.434	4.219	0.039*
rAEF(Median)	0.268	0.377	0.317	1.501	0.587
rAEF(Mean)	0.297	0.279	0.352	0.604	0.485
rAEF(0.1)	0.116	− 0.035	0.592	0.546	0.351
rAEF(0.25)	0.128	0.471	0.378	2.857	0.513
rAEF(0.5)	0.271	0.378	0.311	1.376	0.758
rAEF(0.75)	0.354	1.258	0.372	6.397	1.000
rAEF(0.9)	1.868	1.292	7.931	6.025	0.855

^*^Statistically significant difference between the two groups (P < 0.05).

For the difference between parameters and normal liver parenchyma, ΔPVP including ΔPVP(Min), ΔPVP(Median), ΔPVP (Mean), ΔPVP(0.1), ΔPVP(0.5), ΔHPI (Max) and ΔAEF(Min) were significantly different between the two groups (P<0.05). Among them, all of theΔPVP value andΔAEF value were significantly higher in MVI negative group than those in positive group, while forΔHPI (Max), the value of MVI positive group was significantly higher than that of negative group (Table 3).

Regarding relative parameters, there were statistical differences between the two groups in the parameters of rPVP(Min), rHPI(Max), rAEF(Min) and rAEF(Max). The value of rPVP(Min) and rAEF(Min) in MVI negative group were significantly higher than those in positive group, while for rHPI (Max) and rAEF(Max), the value of MVI positive group was significantly higher than that of negative group (Table 4).

Other parameters had no statistical significance between the two groups (P>0.05). All the HAP related parameters had no statistical significance in predicting MVI, so no relevant values were counted in the Tables 2, 3 and 4.

### Diagnostic performance of the perfusion parameters

**Figure 4:**
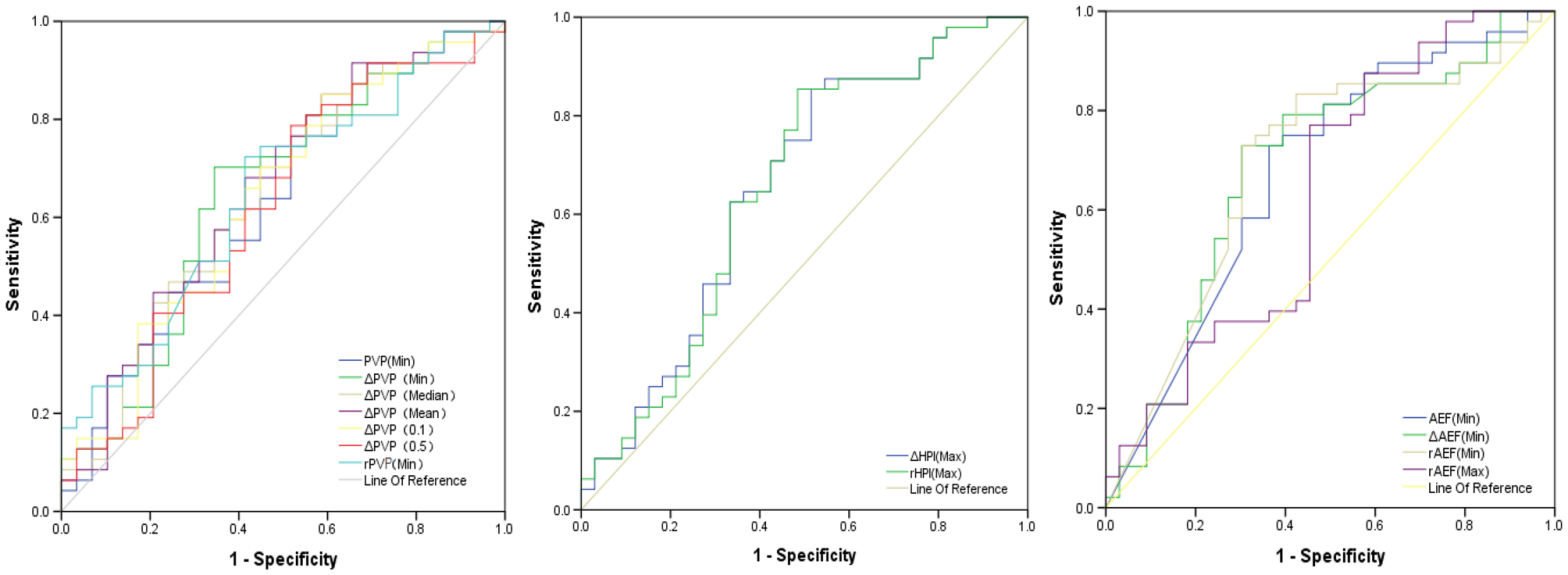
The ROC analysis of the single parameter. The ROC curves of PVP, HPI and AEF for MVI negative and positive of HCC. *PVP* portal vein blood supply perfusion, *HPI* hepatic artery perfusion Index, *AEF* arterial enhancement fraction, *AUC* area under the curve.

**Figure 5:**
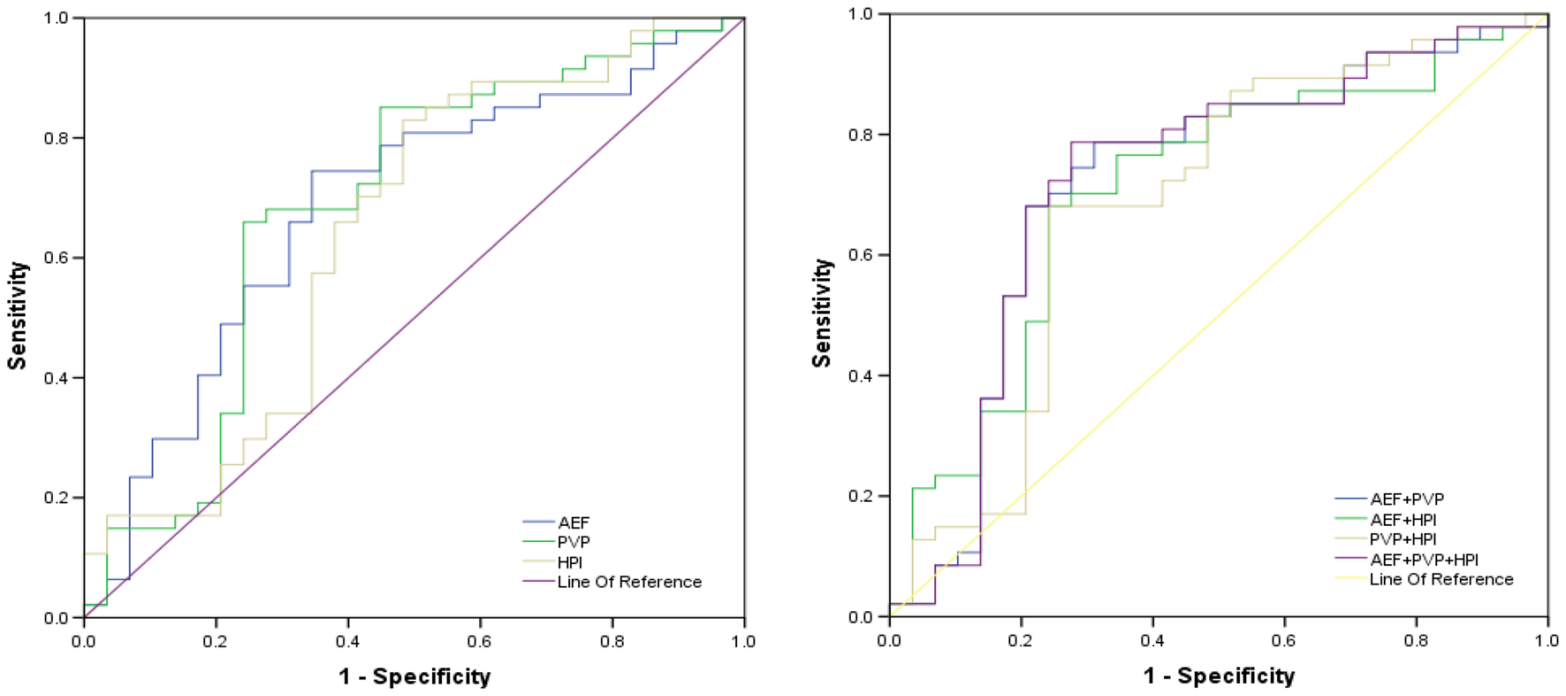
The ROC analysis of the combined parameters. The ROC curves of combined parameters of PVP, HPI and AEF for MVI negative and positive of HCC.AEF refers to the combination of AEF(Min), ΔAEF(Min) and rAEF(Min). PVP refers to the combination ofΔPVP(Min), ΔPVP(Median), ΔPVP(Mean), ΔPVP(0.1), ΔPVP(0.5) and rPVP(Min). HPI refers to the combination of ΔHPI(Max) and rHPI(Max). PVP+HPI refers to the combination of ΔPVP(Mean) and ΔHPI(Max). HPI+AEF refers to the combination of ΔHPI(Max) and rAEF(Min). PVP+AEF refers to the combination of ΔPVP(Mean) and rAEF(Min). PVP+HPI+AEF refers to the combination of ΔPVP(Mean), ΔHPI(Max) and rAEF(Min).

ROC curves were used to evaluate the diagnostic effectiveness of the perfusion parameters in MVI prediction. AUC values, specificity, sensitivity and cut-off values are shown in Table 5, Figs. 4 and 5. As shown in Table 5, the combination of ΔPVP(Mean), ΔHPI(Max) and rAEF(Min) had the highest diagnostic efficacy, with AUC value of 0.741, sensitivity of 0.787 and specificity of 0.742. The two parameters related to HPI (ΔHPI (Max) and rHPI (Max)) had the highest sensitivity of 0.854, while the combination of PVP related parameters had higher specificity of 0.774, AUC value of 0.707, and sensitivity of 0.660.

**Table 5:** ROC analysis of the single and combined parameters.

Variables	AUC	Sensitivity, 100%	Specificity, 100%	Cutoff value
PVP(Min)	0.630	0.745	0.483	0.060
ΔPVP(Min)	0.647	0.702	0.655	− 0.022
ΔPVP(Median)	0.642	0.702	0.552	− 0.054
ΔPVP(Mean)	0.657	0.681	0.586	− 0.016
ΔPVP(0.1)	0.636	0.851	0.414	− 0.071
ΔPVP(0.5)	0.618	0.787	0.483	− 0.067
rPVP(Min)	0.645	0.723	0.586	− 0.720
ΔHPI(Max)	0.654	0.854	0.485	0.035
rHPI(Max)	0.652	0.854	0.515	0.082
AEF(Min)	0.675	0.729	0.636	0.300
ΔAEF(Min)	0.683	0.729	0.697	− 0.073
rAEF(Min)	0.687	0.729	0.697	− 0.281
rAEF(Max)	0.629	0.771	0.545	0.663
PVP	0.707	0.660	0.774	0.657
HPI	0.654	0.813	0.518	0.534
AEF	0.711	0.750	0.667	0.529
PVP+HPI	0.706	0.681	0.773	0.676
HPI+AEF	0.727	0.771	0.667	0.535
PVP+AEF	0.738	0.787	0.710	0.536
PVP+HPI+AEF	0.741	0.787	0.742	0.547

AEF refers to the combination of AEF(Min), ΔAEF(Min) and rAEF(Min). PVP refers to the combination ofΔPVP(Min), ΔPVP(Median), ΔPVP(Mean), ΔPVP(0.1), ΔPVP(0.5) and rPVP(Min). HPI refers to the combination ofΔHPI(Max) and rHPI(Max). PVP+HPI refers to the combination of ΔPVP(Mean) and ΔHPI(Max). HPI+AEF refers to the combination of ΔHPI(Max) and rAEF(Min). PVP+AEF refers to the combination of ΔPVP(Mean) and Raef(Min). PVP+HPI+AEF refers to the combination of ΔPVP(Mean), ΔHPI(Max) and rAEF(Min).

## Discussion

More and more studies have shown that liver cancer with microvascular invasion requires a larger resection range and a wider ablation margin, and should be more cautiously used as a candidate for liver transplantation. As a pathological concept, microvascular invasion is more clinically instructive on how to make accurate prediction before surgery. Through accurate prediction of preoperative MVI, more reasonable treatment options can be selected.

MVI is a nest of malignant cells only visible under a microscope in vessels, including arteries, hepatic vein, and portal vein lined with endothelial cells. The vascular lumen is mainly composed of portal vein branches adjacent to cancer. The presence of cancer cell nests in blood vessels, whether in arteries, hepatic veins, or portal veins, theoretically leads to changes in blood flow perfusion within the lesion. Therefore, our research is based on this hypothesis.

As mentioned above, accurate preoperative diagnosis of MVI remains challenging, but is critical to guide therapeutic options. This study used perfusion parameters from conventional three-phase CT scans to help predict MVI preoperatively and showed promising results.

In the past, many scholars have done a lot of research on preoperative prediction of MVI and achieved good results, especially in terms of clinicoradiological features and radiomics. However, clinicoradiological features of the cancer lesions have the general short-comings of subjective interpretation. Radiomics improved on this front, but still has its own limitations. The reliability and reproducibility of some radiomics features needs to be further studied and clarified, while some other features are difficult to understand by existing medical knowledge. Furthermore, radiomics analysis demands high image quality, which can be significantly affected by scanning conditions and artifacts.

PCT was first conceptualized by Miles et al. and its usage was further broadened due to the emergence of dual maximum slope model proposed by Blomley et al.^21,23^. Based on the special characteristics of dual blood supply of the liver, PCT can achieve quantitatively detection blood perfusion in the lesions, thus providing information regarding biological features of the tumor^24^. It has been proved that tumor blood perfusion parameters can be calculated by linear combination of enhancement curves of aorta and portal vein^25^. This means that the radiation dose by volume (CTDIvol) is significantly lower than that of traditional perfusion scanning. MVI is a pathological concept that can be only diagnosed after surgery. However, the existence of MVI seriously affects the prognosis of patients. Therefore, it is of paramount importance to predict MVI before surgery. PCT has been widely used in liver cancer in the past^26–29^. But to our knowledge, there are relatively few studies on whether it can help predict MVI. In this study, we used CT hemodynamics software to analyze the images of a triphasic enhancement CT scan to obtain the hemodynamic parameters of liver tumors.

Our study showed that the presence of MVI affected the perfusion parameters of liver cancer. MVI can change the blood perfusion of the lesions due to invasions of the minute branches of the portal vein. PVP, AFF, HPI and their related parameters had certain value in predicting MVI. In MVI negative group, the minimum values of PVP, AEF and their related parameters (ΔPVP, rPVP and ΔAEF,rAEF) were higher than those in MVI positive group. The maximum values of HPI related parameters (ΔHPI, rHPI) in MVI negative group were significantly smaller than those in MVI positive group. The combination of PVP, HPI and AEF had the highest diagnostic efficacy, with an AUC value of 0.741, sensitivity of 0.787 and specificity of 0.742. The two parameters related to HPI (ΔHPI (Max) and rHPI(Max)) had the highest sensitivity of 0.854, while the combination of PVP related parameters had a higher specificity of 0.774.

Our study showed that the minimum and small values of PVP and its related parameters in MVI negative group were significantly higher than those in MVI positive group, which may be due to the fact that most of the tumor thrombus exists in the small branches of portal vein^30,31^, resulting in the reduction of portal vein blood flow. Zhao et al. believed that there was no significant difference in perfusion related diffusion parameters in hepatocellular carcinoma with or without MVI^32^. The difference between Zhao’s findings and our research results may be due to the fact that their perfusion evaluation of the lesion was jointly affected by hepatic artery, portal vein and hepatic vein, while we calculated each perfusion separately. Because of the heterogeneity of solid tumors, not all tumors have decreased PVP. And statistically significant changes were not found in all PVP related parameters, but only in the minimum and smaller values.

Studies have shown that liver cirrhosis will lead to a decrease in PVP. In order to compensate for the decrease in PVP, arterial liver perfusion will increase, which eventually leads to an increase in HPI^33,34^. Similarly, microvascular invasion of liver cancer will also cause the decrease of PVP, and thus increases HPI as a consequence. Arterial peritumoral enhancement was usually seen in HCC with positive MVI, which reflected excessive perfusion in arterial phase when tumor thrombus exists in small branches of portal vein. Our study showed that the maximum values ofΔHPI and rHPI of HCC with MVI were significantly higher than those without MVI, which is consistent with previous research results by other groups.

AEF, which reflects the HAP, could also be used to predict MVI^26^. In our study, the maximum values of rAEF of HCC with MVI were significantly higher than those without MVI. This could be explained by the fact that blockage of small branches of portal vein decreased portal vein blood flow perfusion, and in return, leaded to excessive perfusion in arterial phase. On the contrary, the minimum values related to AEF, including AEF, ΔAEF and rAEF, were significantly higher in MVI negative group than those in positive group. The following mechanism may be at play to explain the observation. Due to tumor heterogeneity, arterial phase hyperperfusion of the MVI lesions was not sufficient to compensate for the decrease of PVP. Changes of tumor perfusion caused by MVI is a very complex process. MVI can cause vascular reconstruction, reduce the adhesion of vascular endothelial cells, and thus reduce portal vein resistance^32^. These will entail perfusion behavior changes, and further study is need to expand our knowledge in this regard.

We do acknowledge that our research has limitations. First, our study was a retrospective study, and some potential selection bias cannot be ruled out when selecting cases. Second, the patient sample size was adequate but relatively small, and of single center origin. In planned future studies, an increase of sample size and multicenter research will help to validate further the findings of the current study. Third, MVI was not graded in the current study. Studies by other groups showed that different grades of MVI could be closely related to the prognosis of patients^12,35^. With an improved sample size, further analyses of the relationship between perfusion parameters and MVI grades will be guaranteed in future studies. Fourth, the arterial imaging time used in this article was 30–35 seconds, without the use of bolus tracking. In our further research, we will attempt to use bolus tracking to increase the credibility of the research.

In conclusion, our study confirmed that MVI presence changed HCC tumor perfusion. Liver perfusion parameters derived from traditional triphasic CT scans can help predict MVI quantitatively and noninvasively. This can potentially serve as a preoperative biomarker for patients with HCC.

## Data Availability

The authors confirm that data published in the article are available, and raw data supporting the findings will be shared by the corresponding author upon reasonable request.
